# Pooling annotated corpora for clinical concept extraction

**DOI:** 10.1186/2041-1480-4-3

**Published:** 2013-01-08

**Authors:** Kavishwar B Wagholikar, Manabu Torii, Siddhartha R Jonnalagadda, Hongfang Liu

**Affiliations:** 1 Division of Biomedical Statistics and Informatics, Mayo Clinic, Rochester, MN, USA; 2 Department of Radiology, Georgetown University Medical Center, Washington, DC, USA

## Abstract

**Background:**

The availability of annotated corpora has facilitated the application of machine learning algorithms to concept extraction from clinical notes. However, high expenditure and labor are required for creating the annotations. A potential alternative is to reuse existing corpora from other institutions by pooling with local corpora, for training machine taggers. In this paper we have investigated the latter approach by pooling corpora from 2010 i2b2/VA NLP challenge and Mayo Clinic Rochester, to evaluate taggers for recognition of medical problems. The corpora were annotated for medical problems, but with different guidelines. The taggers were constructed using an existing tagging system MedTagger that consisted of dictionary lookup, part of speech (POS) tagging and machine learning for named entity prediction and concept extraction. We hope that our current work will be a useful case study for facilitating reuse of annotated corpora across institutions.

**Results:**

We found that pooling was effective when the size of the local corpus was small and after some of the guideline differences were reconciled. The benefits of pooling, however, diminished as more locally annotated documents were included in the training data. We examined the annotation guidelines to identify factors that determine the effect of pooling.

**Conclusions:**

The effectiveness of pooling corpora, is dependent on several factors, which include compatibility of annotation guidelines, distribution of report types and size of local and foreign corpora. Simple methods to rectify some of the guideline differences can facilitate pooling. Our findings need to be confirmed with further studies on different corpora. To facilitate the pooling and reuse of annotated corpora, we suggest that – i) the NLP community should develop a standard annotation guideline that addresses the potential areas of guideline differences that are partly identified in this paper; ii) corpora should be annotated with a two-pass method that focuses first on concept recognition, followed by normalization to existing ontologies; and iii) metadata such as type of the report should be created during the annotation process.

## Background

Development of Natural Language Processing (NLP) tools generally requires a set of annotated documents in the application domain [1]. The annotations serve as a reference for constructing rule-based NLP systems and as a training corpus to derive machine learning models for concept extraction. However, in the clinical domain, annotated corpora are often difficult to develop due to high cost of manual annotation involving domain experts and medical practitioners, and also due to concerns for patient confidentiality [2]. Due to high demand, such corpora have been recently created with pioneering effort of some research groups and made available to the scientific community to support studies in clinical NLP [3-5].

Availability of the annotated corpora has fostered the application of machine learning algorithms to concept extraction from clinical notes [6,7]. Supervised machine learning taggers that achieve an accuracy of more than 80% have been developed [8,9], given their great success for general English text [10] and biomedical literature [11-13]. These taggers were developed as an alternative to earlier systems that use dictionaries and rules, e.g. MetaMap [14], MedLEE [15], and SymText/MPLUS [16]. However, machine learning methods are sensitive to the distribution of data, such as the distribution of words in the vocabulary and grammar styles, which could significantly affect the portability of a trained machine learning system across institutions and, thus the value of annotated corpora.

Given the barriers for preparing a large annotated corpus in individual institutions, consolidation of annotation efforts has the potential to advance clinical NLP. One way of leveraging existing efforts is to pool annotated corpora across institutions. Pooling of the annotations to train machine learning taggers may increase performance of the taggers [17]. However there has been little research on associated issues. In this paper we have investigated whether pooling of similar corpora from two different sources can improve performance of resultant machine learning taggers for medical problem detection. We hope that our study will be a useful guide for facilitating reuse of annotated corpora across institutions.

### Pooling Biomedical Corpora

There have been similar efforts to pool corpora in the biomedical domain. Johnson et al. [18] semi-automatically changed the format of the Protein Design Group corpus into two new formats (WordFreak and embedded XML), without altering the semantics, to increase the usage of the corpus. The process of altering the format without change in the semantics was called ‘re-factoring’. Ohta et al. [19] extended the annotation of their GENIA corpus to integrate the annotation style of the GENTAG corpus, which is the other prominent and widely used biomedical corpus, so that their corpus can been pooled with others following the same format. As an extension of this work, Wang et al. [20] pooled these corpora (and a third known as AIMED) [21] hoping to achieve better performance using the large corpus. However, the performance dropped by 12%. Subsequently they analyzed incompatibilities among the corpora. After removing the incompatibilities, they obtained promising performance using the pooled corpora [22]. Recently, Huang et al. [23] have reported significant performance improvement of machine learning based part-of-speech (POS) taggers, by training them on pooled dataset.

We have used publicly available resources, such as the UMLS MetaThesaurus as a term dictionary, GENIA tagger for POS tagging, programs in Mallet machine learning software suite for sequence tagging and clinical corpora from the i2b2/VA challenge, to investigate the feasibility of pooling annotations for clinical concept extraction. Our current effort can potentially benefit research on development of clinical taggers at healthcare institutions, by facilitating use of annotated corpora from other institutions. In the next subsection, we briefly explain the process of annotation for readers who are new to this field.

### Annotation of clinical text

Machine learning based taggers for detecting phrases that convey particular concepts, requires the availability of reports that have been manually marked (annotated) for the phrases. For instance, in the sentence “The patient denies any abdominal pain”, the phrase denoting a medical problem has been marked by the underline. The exercise to manually create such a set of marked reports is initiated with the development of a guideline, which defines what to mark and also how to mark. A group of human annotators then independently follow the guideline to carry out the annotation exercise.

Researchers developing a machine learning tagger at an institute have the option of training the tagger on i) the in-house set of reports that have been manually annotated, ii) reports annotated at another institution or iii) a pooled set constructed by combining i and ii. While the development of the in-house corpus requires several hundred hours of human effort and the associated expenses, the corpus from other institutions may not be portable. In this paper, we have examined the factors associated with the use of corpus from other institutions.

### Overview of current work

We trained and tested taggers on a corpus from Mayo Clinic Rochester [24] and a corpus from the 2010 i2b2/VA NLP challenge [25], and examined the effect of pooling the corpora [26]. These corpora share the property that they were annotated for the same task of developing taggers for detecting medical problems. However the corpora were constructed with different annotation guidelines. The experiments were carried out using an existing machine learning-based tagging system, MedTagger [9], that participated in the 2010 i2b2/VA NLP challenge. In an earlier study, we had reported performance gain for machine learning taggers by pooling corpora across institutions and report types [17]. The corpora used in that study were subsets of the i2b2 corpus and were annotated with the same annotation guideline.

## Results

**Figure 1:**
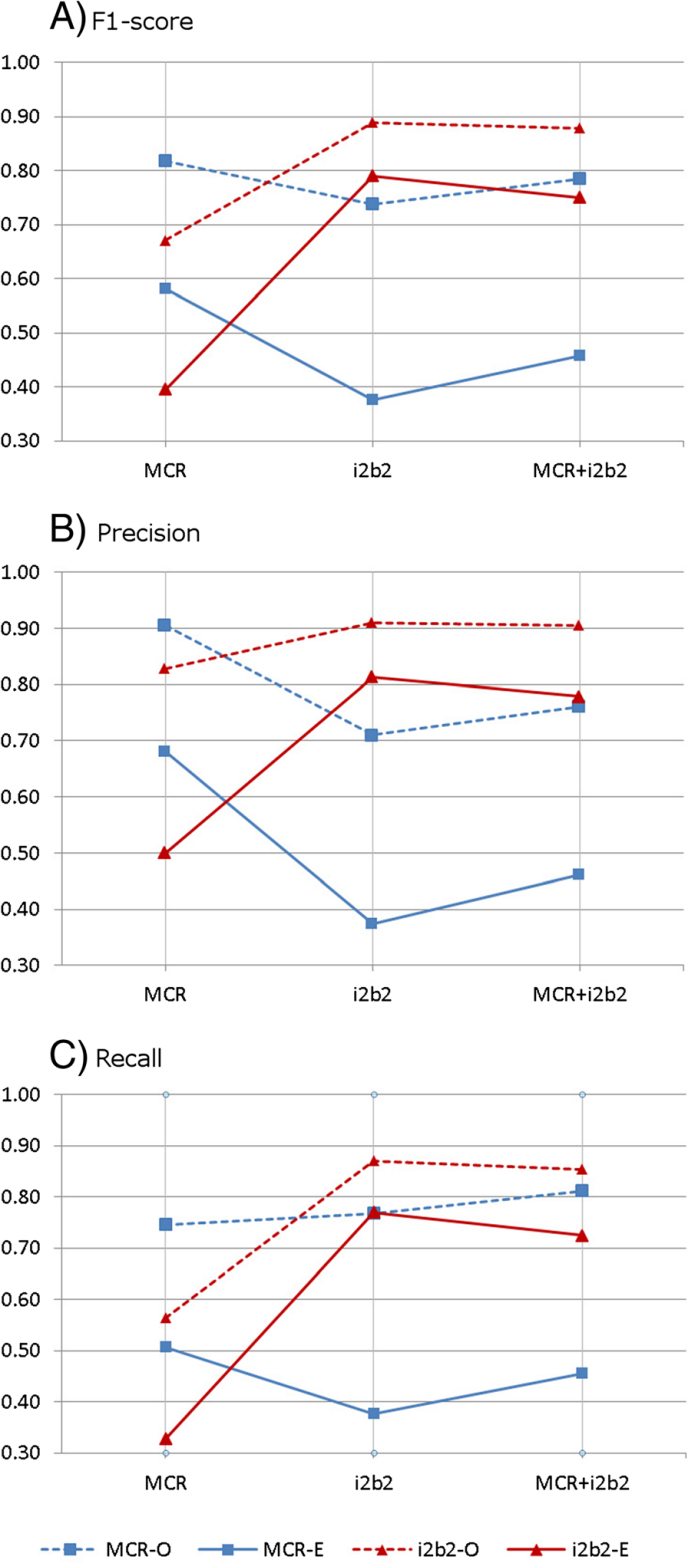
**Performance measures of the taggers.** The plots **A**, **B** and **C** show the F1-score, precision and recall respectively. Each line in the figure corresponds to the test set (MCR or i2b2) and the evaluation method: Exact (E) with solid lines or Overlap (O) with dashed lines. The horizontal axis indicates the training sets: MCR, i2b2 and combined (MCR + i2b2).

Figure 1 summarizes the results of the experiments. Detailed results are tabulated in the Additional file 1.

### Intra-corpus testing

Taggers performed the best when the training and test sets were from the same corpus. F1-scores for MCR and i2b2 cross-validations were 0.58 and 0.79 for ‘exact span’ evaluation, respectively. The higher F1-scores for i2b2 as compared to MCR could be due to a lower diversity of report types and annotations. Addition of more reports to MCR corpus might lead to improved performance for MCR. For the ‘overlap spans’, the F1-scores were 0.82 for MCR and 0.89 for i2b2. The performance patterns were similar for recall and precision for the exact and overlap span evaluations.

### Inter-corpora testing

Performance of the taggers was poor when they were trained exclusively on reports from the other corpora. For tagger trained on i2b2 and tested on MCR, the F1 score was 0.38 for exact spans. Similarly for tagger trained on MCR and tested on i2b2, the scores was 0.40.

Supplementation of the training set with reports from other corpora decreased the performance, by 12% points for MCR and 4% points for i2b2. The greater degradation for MCR is likely due to small size of the corpus as compared to the i2b2 corpus, i.e. the proportion of data supplemented to MCR training set was much larger than that for i2b2. The pattern was similar for precision and recall, and for the ‘overlap span’ evaluation. An exception was the improvement in the recall on MCR corpus when it was supplemented with i2b2 corpus, using ‘overlap span’ evaluation.

Results suggest that the corpora are incompatible for simple pooling. In an earlier study we had reported performance gain for machine learning taggers by pooling corpora across institutions and report types [17]. The corpora used in that study were subsets of the i2b2 corpus and were annotated with the same guideline. Contrastingly, in the current study, the corpora differ in their annotation guidelines, and also have different distributions of report types. Also the corpora sizes are different from the ones examined in the earlier study. Hence we investigated the effect of differences in the annotation guidelines, distributions of report types and corpora sizes, on the performance of taggers trained on pooled corpora, as described in the following sub-sections.

### Guideline differences

We examined the annotation guidelines to identify factors that contributed to the performance degradation of taggers trained on pooled corpora.

#### Concept definition

Annotation guidelines for the two corpora differed slightly in definition of concepts. i2b2 annotation guideline extends definition of medical problem beyond the semantic type of signs/symptoms and disease/syndrome (disorder), to include pathologic functions, mental dysfunction, molecular dysfunction, congenital abnormality, acquired abnormality, neoplastic process, and virus/bacterium. It also allows the annotator to mark medical problems not covered in the UMLS.

MCR annotation guideline defined signs/symptoms and disorders, which we mapped to the problem class. Signs/symptoms were defined as concepts that mapped to SNOMED-CT subset of semantic type signs/symptoms. Disease/syndrome had a looser definition that extended beyond the semantic types for ‘i2b2 medical problem’, to include injury or poisoning, behavioral dysfunction, cell dysfunction, experimental model of disease and anatomical abnormality but excluded virus/bacterium.

#### Articles

**Figure 2:**
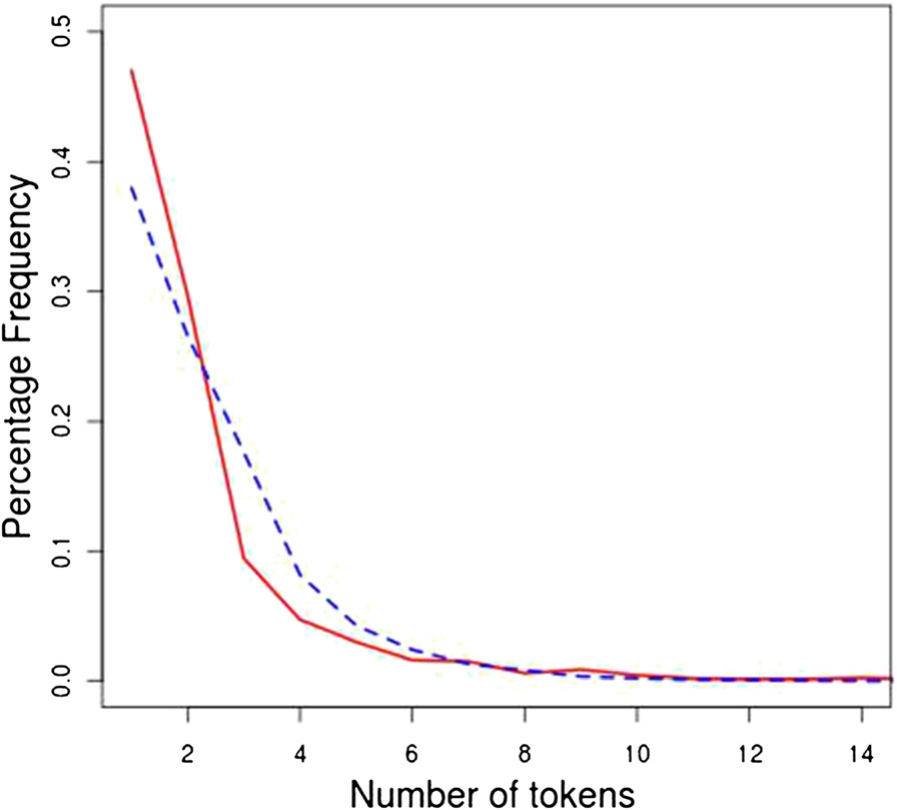
**Distribution of number of tokens per annotation in the two corpora.** MCR annotations (red line) are shorter than the i2b2 annotations (blue dashed line).

The i2b2 annotations included articles, e.g. “the cough” and “a fever”, while MCR annotations did not. Nearly 11% of the i2b2 annotations began with an article (Table 1). This contributes to the generally longer length of the i2b2 annotations (Figure 2).

**Table 1:** Frequency of i2b2 annotations that begin with an article

**Article**	**Frequency**
a	562
the	269
any	154
some	134
an	126
this	25

#### Possessive pronouns

i2b2 annotations included possessive pronouns, e.g. “his cancer”, while MCR annotations did not. 3% of i2b2 annotations began with ‘his’ (174) or ‘her’ (200).

#### Concepts not pertaining to patients

MCR annotations included concepts that were not pertaining to patients. For instance, disease names in organizational unit names, e.g. “cancer” in “cancer department”. Concepts that are not directly related to the patient were not annotated in i2b2 corpus.

#### Prepositional phrases

The i2b2 guidelines specify that one prepositional phrase following a concept can be included in the annotation if it does not contain a markable concept and/either indicates an organ/body part. Also a preposition can be included in a concept phrase if words therein can be rearranged to express the same concept without the preposition. For example, ‘pain in chest’ is a viable concept phrase according to the i2b2 guidelines schema because it indicates body part and can be rephrased without ‘in’ as ‘chest pain’. In contrast, the text segment ‘removal of mass’ is annotated as two concept phrases as it cannot be rearranged to express the same concept without the preposition. MCR guidelines did not explicitly address this issue.

#### Conjunctions

The i2b2 guidelines specify that conjunctions that denote lists are included if they occur within the modifiers or are connected by a common set of modifiers. If the portions of the lists are otherwise independent, they should not be included. For example, the text segment ‘metastases in the liver and pancreas’ is a valid concept phrase including ‘and’, while the segment ‘diarrhea, nausea and vomiting’ is not valid. The latter is annotated as three concept phrases. MCR guidelines did not explicitly address this issue.

### Reconciliation of annotations differences

**Figure 3:**
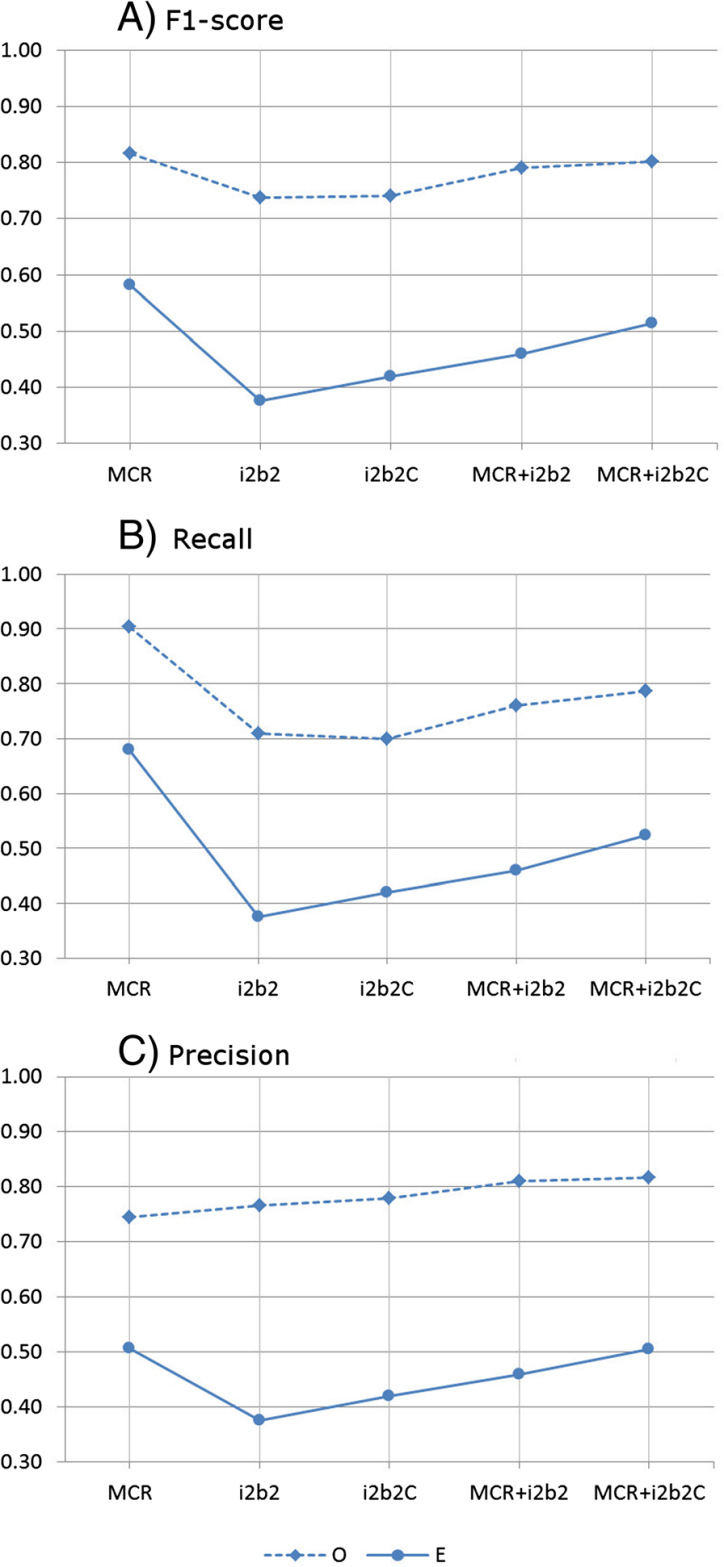
**Performance measures of the taggers trained on the curated i2b2 corpus and tested on MCR corpus.** The plots **A**, **B** and **C** show the F1-score, precision and recall respectively. There are two lines in each plot that correspond to the two evaluation methods: Exact (E) with solid lines and Overlap (O) with dashed lines. The horizontal axis indicates the training sets: i2b2, i2b2C (curated), MCR + i2b2 (combined) and MCR + i2b2C (MCR combined with curated i2b2).

To investigate the effect of annotation differences due to the differing guidelines, we considered curation of the annotations. Rectification of the differences in all guideline factors would require considerable manual effort. Hence, we restricted our effort to automated rectification of a subset of the factors. Specifically, we removed articles and possessive pronouns from i2b2 annotations. Fifteen percent of the i2b2 annotated phrases were modified. When this partially rectified corpus was used to supplement training data for the MCR corpus, there was lesser degradation of the performance measures. The F1-score degraded by 6% points instead of 12% points for exact match and 2% points instead of 3% points for overlapping match (Figure 3 and Additional file 1).

Table 2 shows the overlap in the annotated phrases in the two corpora. Before rectification of the annotation differences, 42.9% of MCR annotations exactly matched i2b2 annotations, i.e. from the 2,076 concept annotations in MCR, there were 890 annotations that exactly matched with an i2b2 annotation. When one start word was ignored 55.1% of the annotations matched and when one word was ignored 55.7% matched. After the i2b2 corpus was curated to partially rectify the annotation differences, there was an improvement in the overlap of the corpora, as shown in Table 2.

**Table 2:** Overlap of annotations in the corpora

**Annotation set**	**Exact match**	**Ignoring one start word**	**Ignoring one word**
MCR	42.9/43.7	55.1/55.7	56.0/56.7
i2b2	22.6/25.2	32.6/33.6	33.6/34.7

Each cell in the table gives the percentage of annotations that matched with the other corpus before and after automated curation of the i2b2 corpus to partially rectify the annotation differences.

The annotations in MCR corpus were mapped to the highest level of granularity-- to the UMLS CUIs. The MCR annotations were restricted strictly to the UMLS. Consequently, these annotations can be expected to inherit the limitations of UMLS which includes lack of concept coverage. This would possibly be the reason why the taggers trained on MCR corpus and tested on i2b2 corpus showed greater degradation in performance than vice-versa. The annotation guideline for the i2b2 corpus advocated a more intuitive approach for annotation. I2b2 annotators used UMLS definitions to guide the annotation, which allowed them greater flexibility to annotate and even include phrases that were not covered in the UMLS.

Hence, to facilitate reuse of the annotations for developing machine learning models for concept recognition, we suggest the following two-step approach for annotation. Annotators should first mark the phrases that correspond to the concept of interest (perform concept recognition), and then normalize the annotations by mapping each annotation to the set of ontology nodes with the highest possible granularity. When normalization is not possible to any ontology node, the phrase should be marked as a ‘novel’ concept.

For developing machine learning applications it is critical that all the phrases that map to the concept of interest are annotated, by ensuring that even those which are not covered in the reference ontology are marked up. The first pass of ‘concept recognition’ would ensure that all the concepts are covered. The second pass of ‘normalization’ will facilitate the filtering/sub-classing of annotations for developing machine learning taggers for a particular sub-class. This two-pass annotation method will facilitate the pooling of corpus with other similar corpora. Also the ‘novel’ annotation class will be useful for adding new ontology terms.

### Report type

In addition to the differences in the annotation guidelines, the performance of the taggers could be affected by the distribution of report types in the corpora. i2b2 corpus included discharge summaries and progress notes, while MCR corpus had a wider variety, since the reports were randomly selected from the EMR system for annotation. Named entities may vary in their distributions on report types. For instance the history and examination reports will have a high density of patient symptoms as compared to the progress notes that will mainly refer to the symptoms addressed by the current treatment. Also the progress notes will perhaps contain more medical terminology instead of ordinary English words reported by the patient in the history and examination reports. The distribution of the medical terminologies on the report types may also depend on the institution, as many institutions have their own report formats. However the reports in either corpus did not have meta-data about the type of report. Hence, the authors could not investigate the ‘report-type’ factor further.

### Corpus size

**Figure 4:**
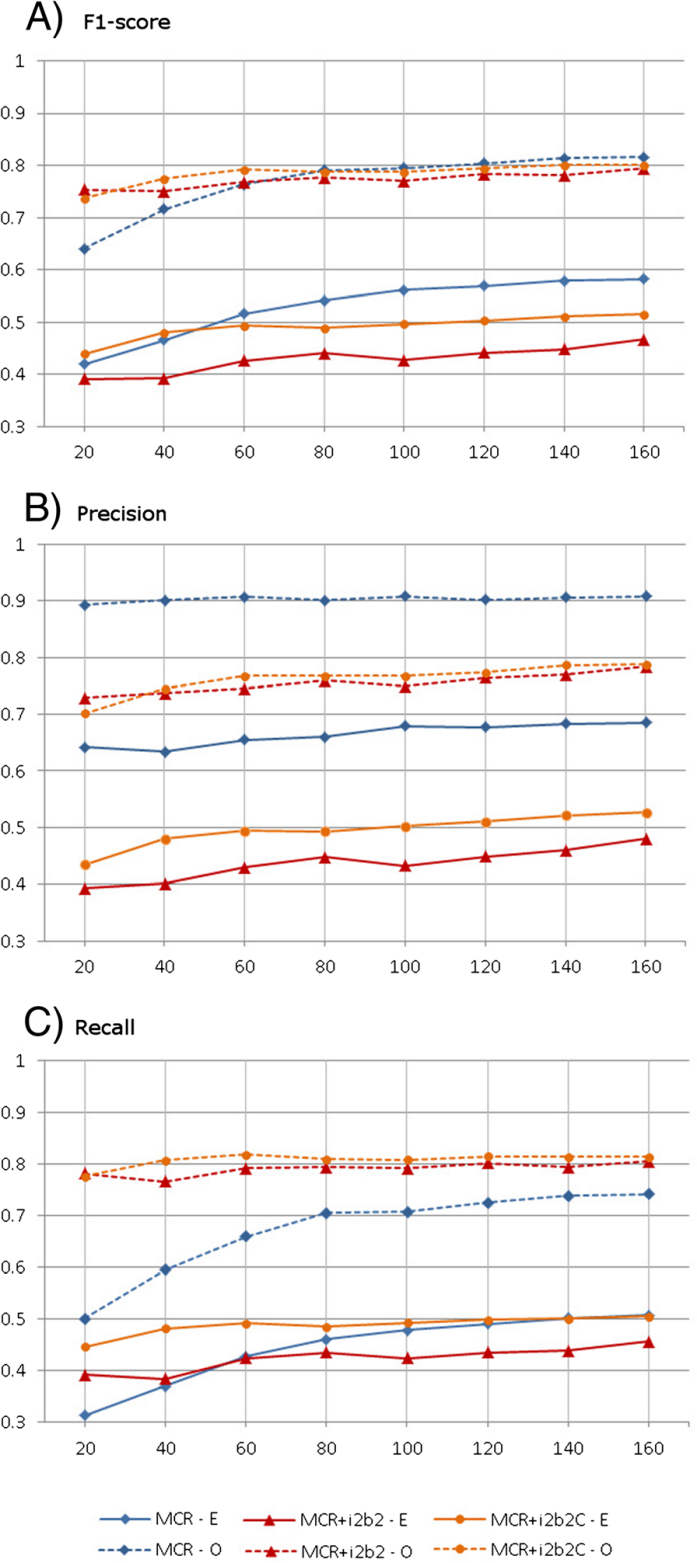
**The plots A, B and C show the F1-score, precision and recall of the taggers respectively.** The horizontal axis indicates the size of the MCR subsets used in the cross-validation. In each plot the colors of the horizontal lines correspond to the corpus used in the cross-validation experiment, viz. MCR, MCR + i2b2 (MCR with training fraction supplemented by i2b2) and MCR + i2b2C (MCR with training fraction supplemented by curated i2b2). The two types of evaluation methods are represented using different line styles: Exact (E) with solid lines and Overlap (O) with dashed lines.

To examine the effect of size, we measured the tagger performance on subsets of MCR corpus of various sizes, by performing 5 fold cross-validation experiments. This was compared to the performance after pooling the MCR subsets with the original and curated i2b2 corpus for training, i.e. the i2b2 corpus was used to supplement the training fraction of the MCR corpus during the cross-validations. Each experiment was repeated 5 times and the average performance measures were computed, i.e. 5 times 5-fold cross-validation was performed. We had increased the runs for cross-validating the subsets from 3 to 5. The subsets were smaller in size, which increased the variation of the accuracy measurements. The additional runs were required to compensate for the increase in variation, so as to provide adequate confidence of the accuracy measurements. The results are summarized in Figure 4 and tabulated in the Additional file 1.

The performance of taggers trained and tested on MCR corpus increases in F1-score and recall as the corpus size increases. The increase is rapid at first with increment in the corpus size, but later forms a plateau. The precision, on the other hand, is nearly unaffected by the corpus size.

Pooling with the i2b2 corpus nearly always increases the recall. However as the precision always degrades on pooling, the F1-score first increases with pooling and then degrades. A possible explanation is that the smaller subsets of MCR corpus are deficient in all the annotation patterns, and when these are supplemented by the i2b2 curated corpus, there is an improvement in recall.

The improvement in recall for smaller sizes of MCR subsets is greater than the degradation in precision that occurs due to pooling, which translates to an improvement in F1-score. When the MCR subset size crosses a threshold, the improvement in recall cannot surpass the degradation in precision, which lowers the F1-score. This result leads to a hypothesis that pooling with a compatible corpus from another institution may be beneficial and an economically favorable alternative to extending the in-house annotated corpus, only when the in-house corpus is below a critical size. However our analysis is limited to a single observation and further studies on other corpora are needed to investigate the combined effect of differences in the annotation guidelines, distributions of report type and sizes.

The tagger performance with the curated i2b2 corpus was greater than with the original corpus for all subsets of MCR corpus. The simple approach of automated annotation, described earlier increased the threshold of the MCR subset size where the F1-score dips on pooling.

### Summary

In summary, simple pooling of corpora was overall found to reduce the tagger performance. We examined the annotation guidelines for the corpora to delineate several inconsistencies that include concept definition, articles, possessive pronouns, unrelated concepts, prepositional phrases and conjunctions. Rectification of a subset of the annotation differences using an automatic approach reduced the performance degradation that occurred on pooling. The effect of distribution of report types could not be studied as the corpora were not annotated for report type. The effect of pooling was found to depend on the corpus size, as pooling was found to improve tagger performance for smaller subsets of the MCR corpus. This result suggests that pooling with corpora from another institution may be beneficial when the in-house corpus is below a critical size. Further studies on different corpora are needed to elucidate the relationship between the above mentioned factors and performance of taggers trained on pooled corpora. The investigation of these relationships would be a useful guide for researchers to develop machine learning taggers for clinical text.

## Conclusions

We investigated whether pooling of corpora from two different sources, can improve performance and portability of resultant machine learning taggers. The effect of pooling local and foreign corpora, is dependent on several factors that include compatibility of annotation guidelines, distribution of report types and corpus size. Simple automatic methods to rectify some of the guideline differences can be useful to facilitate pooling. Practically useful machine taggers can be possibly developed by annotating a small local corpus, and pooling it with a large similar corpus, available for reuse from another institution. The benefits of pooling diminish as more local annotations are created. Our findings need to be confirmed with further studies using different corpora and machine taggers.

### Future directions

Studies on different corpora are needed to elucidate the relationship between the above mentioned factors and performance of taggers trained on pooled corpora. We plan to investigate whether weighting of features for the machine learning tagger and filtering of the annotations using a dictionary lookup, can improve the tagger performance on pooling the corpora. Another interesting direction of investigation would be to train machine taggers separately on local and foreign corpora and then to combine the taggers using machine learning.

We suggest that future initiatives for clinical annotations should consider guideline factors delineated in this paper for development of the annotation guidelines, so that their annotation effort can be utilized by others. Moreover the guidelines should also include instructions to annotate metadata about the reports, so that reports of the same type from different corpora can be readily pooled for enhancing machine learning based taggers. The authors are aware of an effort in this direction [27], but there needs to be consensus for wider utilization of the standard for annotation of new corpora. The annotation groups involved in clinical research should come together to develop a standard annotation guideline that facilitates reuse of annotation efforts. We also suggest a two-pass annotation method that focuses first on concept recognition, followed by normalization to existing ontologies, to facilitate the pooling of corpora.

## Methods

### Corpora

Two annotated corpora were used in this study (Table 3). The corpora differed in their sources as well their annotation guidelines, but contained annotations for the same concept type, i.e. ‘medical problems’.

**Table 3:** Summary statistics for the corpora

**Set name**	**Documents**	**Lines**	**Tokens**	**Concepts**	**% of tokens included in concept annotation**
i2b2/VA	349	30,673	260,570	11,967	10.9
Mayo	160	2,487	40,988	2,076	11.3

The first corpus consisted of 349 clinical reports from the ‘2010 i2b2/VA challenge on concept assertions and relations in clinical text’ [25] that were provided to the participants for training. Partners Healthcare, Beth Israel Deaconess Medical Center and University of Pittsburgh Medical Center contributed discharge summaries for this corpus, and University of Pittsburgh Medical Center also contributed progress reports. This corpus was annotated for patient medical problems (signs/symptoms and disorders), treatments and tests.

The second corpus had 160 clinical notes from Mayo Clinic Rochester (MCR) [24,28]. These were annotated for signs/symptoms, disorders, medications and procedures. The annotation class ‘problem’ from the first corpus was equivalent to the combination of classes -- signs/symptoms and disorders in the second corpus and we carried out experiments with reference to this class.

### MedTagger

The experiments reported in this paper were carried out using an existing tagging system, MedTagger that participated in the 2010 i2b2/VA NLP challenge. This is an adaptation of the BioTagger-GM system that was originally developed to identify gene/protein names in biomedical literature [9]. The pipeline of this system consisted of dictionary lookup, part of speech (POS) tagging and machine learning for named entity prediction and concept extraction.

#### Dictionary lookup

We used the UMLS MetaThesaurus [29] and a collection of words used in a clinical vocabulary viewer [30] as the domain dictionary. The input text and dictionary were normalized to facilitate flexible matching. The normalization process included (a) converting words to base form using the UMLS SPECIALIST lexicon, (b) changing letters to lower case, (c) removing punctuation symbols, and (d) converting digits and Greek letters to 9 and ‘G’ respectively. The dictionary lookup tagged all phrase occurrences, including overlapping phrases.

#### POS tagging

We used GENIA tagger for labeling parts of speech to all input tokens. GENIA tagger [31] is based on maximum entropy models trained on biomedical text as well as generic English text.

#### Machine learning

Using the dictionary lookup and POS tagging results, we derived a set of features for each token. These were collated with other commonly used features for named entity recognition, such as words, word affixes and word shapes. The features were fed to a sequence tagger using a conditional random field (CRF) model [32] with a token window size of five.

### Experiment

**Figure 5:**
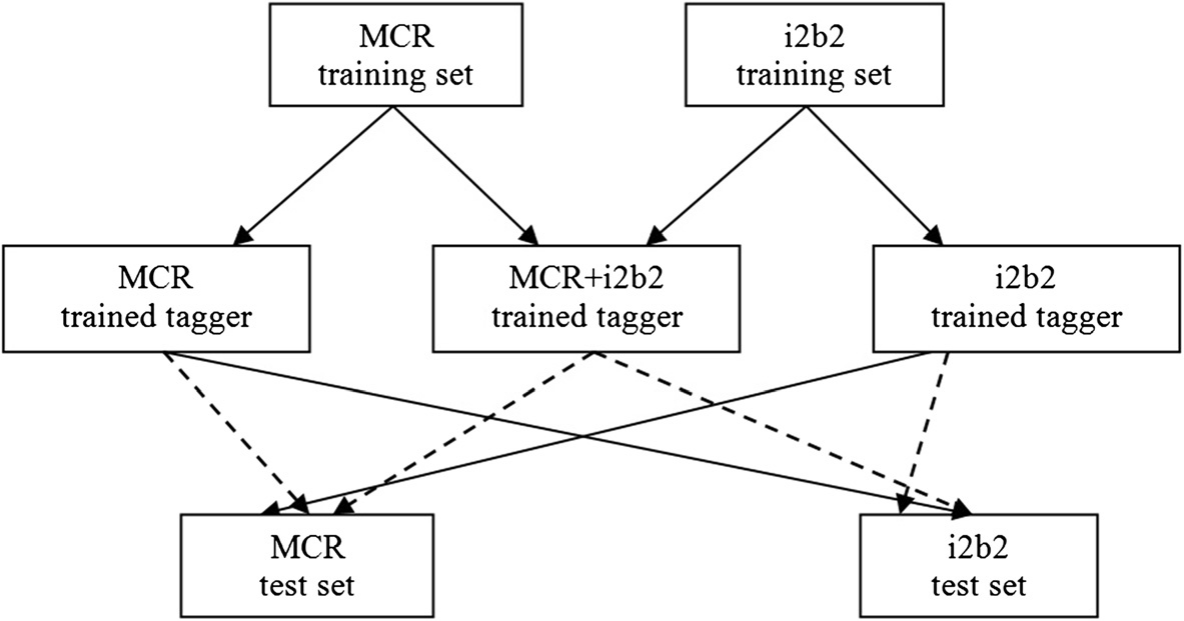
**Design for different training/testing experiment reported in this paper.** Two types of arrows point to the test sets: **a**) Solid lines representing a direct evaluation on the test set, **b**) The dotted lines represent 5 fold cross-validation in which the fraction of the corpus being tested is excluded from the training set.

We designed our experiments to examine effect of using pooled training sets on the performance of machine learning taggers for concept extraction (Figure 5). The taggers were trained to recognize medical problems, including signs/symptoms and disorders. First, we trained the tagger on i2b2 corpus and tested it on MCR corpus and vice versa. We then performed 5-fold cross validation experiments on MCR, i2b2 and the combined (i2b2 + MCR) corpora. We repeated the cross-validation on MCR corpus after supplementing the training fraction with the i2b2 corpus during each of the cross validation runs. This design was repeated for the i2b2 corpus by using MCR corpus to supplement the training. The cross-validation experiments were repeated three times to average the performance scores, i.e. 3 times 5-fold cross-validation was performed.

Two kinds of evaluations were preformed: ‘exact span’ matching and ‘overlap span’ matching. In ‘exact span’ matching, the annotations were counted as matches if begin and end spans matched. In case of ‘overlap span’ evaluation the annotations were counted as matches, if there was any overlap in the span ranges. The overlap span is a more lenient measure of performance. It was expected to be more useful in this study, because a wide variation of phrases was anticipated between the corpora, as the corpora originated from different institutions and used different annotation guidelines.

Performance measures of precision, recall and F1 score were computed for the experiments (Figure 1). The measures are defined as follows:

$$\text{Precision}=\text{true}\;\text{positives}/\left(\text{true}\;\text{positives}+\text{false}\;\text{positives}\right)$$

$$\text{Recall}=\text{true}\;\text{positives}/\left(\text{true}\;\text{positives}+\text{false}\;\text{negatives}\right)$$

$$F1=2\times \left(\text{Recall}\times \text{Precision}\right)/\left(\text{Recall}+\text{Precision}\right)$$

## Abbreviations

NLP: Natural language processing; EMR: Electronic medical record; MCR: MAYO clinic Rochester; CRF: Conditional random field; POS: Part of speech.

## Competing interests

The authors declare that they have no competing interest.

## Authors’ contribution

KW carried out the experiments, led the study design and analysis and drafted the manuscript. MT helped with the experiments, participated in the study analysis and manuscript drafting. SJ participated in the study analysis and manuscript drafting. HL conceived the study, helped with the experiments, participated in the study design and analysis, and drafting of the manuscript. All authors read and approved the final manuscript.

## Supplementary Material

Additional file 1 **Appendix.** Tables detailing the figures included in the paper. Click here for file

## References

[B1] Demner-Fushman D Chapman WW McDonald CJ What can natural language processing do for clinical decision support? J Biomed Inform 2009 42 5 760 772 10.1016/j.jbi.2009.08.007 19683066 PMC2757540

[B2] Meystre SM Savova GK Kipper-Schuler KC Hurdle JF Extracting information from textual documents in the electronic health record: a review of recent research Yearb Med Inform 2008 1 128 144 18660887

[B3] Uzuner O Luo Y Szolovits P Evaluating the state-of-the-art in automatic de-identification J Am Med Inform Assoc 2007 14 5 550 563 10.1197/jamia.M2444 17600094 PMC1975792

[B4] Uzuner O Goldstein I Luo Y Kohane I Identifying patient smoking status from medical discharge records J Am Med Inform Assoc 2008 15 1 14 24 17947624 10.1197/jamia.M2408 PMC2274873

[B5] Uzuner O Solti I Cadag E Extracting medication information from clinical text J Am Med Inform Assoc 2010 17 5 514 518 10.1136/jamia.2010.003947 20819854 PMC2995677

[B6] Wang Y Patrick J Cascading classifiers for named entity recognition in clinical notes Proceedings of the workshop on biomedical information extraction 2009 Borovets, Bulgaria. 1859783: Association for Computational Linguistics 42 49

[B7] Li D Kipper-Schuler K Savova G Conditional random fields and support vector machines for disorder named entity recognition in clinical texts Proceedings of the workshop on current trends in biomedical natural language processing 2008 Columbus, Ohio. 1572326: Association for Computational Linguistics 94 95

[B8] Jonnalagadda S An effective approach to biomedical information extraction with limited training data (PhD Dissertation, Arizona State University) 2011 Phoenix, Arizona: PhD Phoenix

[B9] Torii M Hu Z Wu CH Liu H BioTagger-GM: a gene/protein name recognition system J Am Med Inform Assoc 2009 16 2 247 255 10.1197/jamia.M2844 19074302 PMC2649315

[B10] Tjong Kim Sang EF De Meulder F Introduction to the CoNLL-2003 shared task Seventh conference on natural language learning 2003 Edmonton, Canada 142 147

[B11] Wilbur J Smith L Tanabe T BioCreative 2 gene mention task Proceedings of the second BioCreative challenge workshop 2007 Madrid, Spain: Proceedings of the second biocreative challenge evaluation workshop Vol: 23 7 16

[B12] Arighi CN Roberts PM Agarwal S Bhattacharya S Cesareni G Chatr-Aryamontri A Clematide S Gaudet P Giglio MG Harrow I BioCreative III interactive task: an overview BMC Bioinformatics 2011 12 Suppl 8 S4 10.1186/1471-2105-12-S8-S4 22151968 PMC3269939

[B13] Kim JD Nguyen N Wang Y Tsujii J Takagi T Yonezawa A The genia event and protein coreference tasks of the BioNLP shared task 2011 BMC Bioinformatics 2012 13 Suppl 11 S1 22759455 10.1186/1471-2105-13-S11-S1 PMC3384256

[B14] Aronson AR Lang FM An overview of MetaMap: historical perspective and recent advances J Am Med Inform Assoc 2010 17 3 229 236 20442139 10.1136/jamia.2009.002733 PMC2995713

[B15] Bakken S Hyun S Friedman C Johnson S A comparison of semantic categories of the ISO reference terminology models for nursing and the MedLEE natural language processing system Stud Health Technol Inform 2004 107 Pt 1 472 476 15360857

[B16] Haug P Koehler S Lau LM Wang P Rocha R Huff S A natural language understanding system combining syntactic and semantic techniques Proc Annu Symp Comput Appl Med Care 1994 247 251 7949928 PMC2247803

[B17] Torii M Wagholikar K Liu H Using machine learning for concept extraction on clinical documents from multiple data sources J Am Med Inform Assoc 2011 18 5 580 587 10.1136/amiajnl-2011-000155 21709161 PMC3168314

[B18] Johnson HL Baumgartner WA Krallinger M Cohen KB Hunter L Corpus refactoring: a feasibility study Journal of Biomedical Discovery and Collaboration 2007 2 1 4 10.1186/1747-5333-2-4 17854502 PMC2072937

[B19] Ohta T Kim J-D Pyysalo S Wang Y Tsujii J Incorporating GENETAG-style annotation to GENIA corpus Workshop on current trends in biomedical natural language processing 2009 Stroudsburg, PA, USA: Association for Computational Linguistics 106 107

[B20] Wang Y Kim J-D Sætre R Pyysalo S Tsujii J Investigating heterogeneous protein annotations toward cross-corpora utilization BMC Bioinformatics 2009 10 1 403 10.1186/1471-2105-10-403 19995463 PMC2804683

[B21] The AIMed corpusftp://ftp.cs.utexas.edu/pub/mooney/bio-data/interactions.tar.gz

[B22] Wang Y Sætre R Kim J-D Pyysalo S Ohta T Tsujii JI Improving the inter-corpora compatibility for protein annotations J Bioinform Comput Biol 2010 08 05 901 10.1142/S0219720010004999 20981894

[B23] Fan JW Prasad R Yabut RM Loomis RM Zisook DS Mattison JE Huang Y Part-of-speech tagging for clinical text: wall or bridge between institutions? AMIA Annu Symp Proc 2011 2011 382 391 22195091 PMC3243258

[B24] Savova GK Masanz JJ Ogren PV Zheng J Sohn S Kipper-Schuler KC Chute CG Mayo clinical text analysis and knowledge extraction system (cTAKES): architecture, component evaluation and applications Journal of American Medical Informatics Assocociation 2010 17 5 507 513 10.1136/jamia.2009.001560 PMC2995668 20819853

[B25] Uzuner O South BR Shen S Duvall SL 2010i2b2/VA challenge on concepts, assertions, and relations in clinical text J Am Med Inform Assoc 2011 18 5 552 556 10.1136/amiajnl-2011-000203 21685143 PMC3168320

[B26] Wagholikar K Torii M Jonnalagadda S Liu H Feasibility of pooling annotated corpora for clinical concept extraction AMIA summit on clinical research informatics 2012 San Francisco, CA: American Medical Informatics Summits on Translational Science Proceedings 63 70 PMC3392069 22779047

[B27] Annotation schema for marking spans of clinical conditions in clinical texthttp://orbit.nlm.nih.gov/resource/annotation-schema-marking-spans-clinical-conditions-clinical-text

[B28] Ogren PVS Guergana K Chute Y Christopher G Constructing evaluation corpora for automated clinical named entity recognition LREC’08 2008 Marrakech, Morocco: Proceedings of the Sixth International Conference on Language Resources and Evaluation LREC'08 3143 3150

[B29] Bodenreider O The unified medical language system (UMLS): integrating biomedical terminology Nucleic Acids Res 2004 32 Database issue D267 270 14681409 10.1093/nar/gkh061 PMC308795

[B30] Friedman C Liu H Shagina L A vocabulary development and visualization tool based on natural language processing and the mining of textual patient reports J Biomed Inform 2003 36 3 189 201 10.1016/j.jbi.2003.08.005 14615228

[B31] Yoshimasa Tsuruoka YT Jin-Dong K Tomoko O John MN Sophia A Junichi T Developing a robust part-of-speech tagger for biomedical text Advances in informatics - 10th panhellenic conference on informatics 2005 Heidelberg, Berlin: Springer Berlin 382 392

[B32] McCallum AK MALLET: a machine learning for language toolkit 2002 http://mallet.cs.umass.edu

