# Supplementary material for: Pooling annotated corpora for clinical concept extraction
Source: J Biomed Semantics. 2013 Jan 8;4:3. doi: 10.1186/2041-1480-4-3 (PMC3599895; doi:10.1186/2041-1480-4-3)
Supplement: Additional file 1 — Appendix. Tables detailing the figures included in the paper. [file 2041-1480-4-3-S1.pdf]

Appendix- table for Figure 1

Measure	Test Set Evaluation	MCR	i2b2	MCR+i2b2
F1	MCR-O	0.82	0.74	0.79
F1	MCR-E	0.58	0.38	0.46
F1	i2b2-O	0.67	0.89	0.88
F1	i2b2-E	0.40	0.79	0.75
Precision	MCR-O	0.90	0.71	0.76
Precision	MCR-E	0.68	0.38	0.46
Precision	i2b2-O	0.83	0.91	0.90
Precision	i2b2-E	0.50	0.81	0.78
Recall	MCR-O	0.74	0.77	0.81
Recall	MCR-E	0.51	0.38	0.46
Recall	i2b2-O	0.56	0.87	0.85
Recall	i2b2-E	0.33	0.77	0.72

Appendix- table for Figure 3

Measure	Eval_type	MCR	i2b2	i2b2C	MCR+i2b2	MCR+i2b2C
F1	O	0.82	0.74	0.74	0.79	0.80
F1	E	0.58	0.38	0.42	0.46	0.52
Precision	O	0.90	0.71	0.70	0.76	0.79
Precision	E	0.68	0.38	0.42	0.46	0.52
Recall	O	0.74	0.77	0.78	0.81	0.82
Recall	E	0.51	0.38	0.42	0.46	0.51

Appendix- table for Figure 4

		MCR		MCR+i2b2		MCR+i2b2C	
**Measure**	**MCR size**	**E**	**O**	**E**	**O**	**E**	**O**
F1	20	0.42	0.64	0.39	0.75	0.44	0.74
F1	40	0.47	0.72	0.39	0.75	0.48	0.77
F1	60	0.52	0.76	0.43	0.77	0.49	0.79
F1	80	0.54	0.79	0.44	0.78	0.49	0.79
F1	100	0.56	0.80	0.43	0.77	0.50	0.79
F1	120	0.57	0.80	0.44	0.78	0.50	0.79
F1	140	0.58	0.81	0.45	0.78	0.51	0.80
F1	160	0.58	0.82	0.47	0.79	0.52	0.80
Precision	20	0.64	0.89	0.39	0.73	0.43	0.70
Precision	40	0.63	0.90	0.40	0.74	0.48	0.74
Precision	60	0.66	0.91	0.43	0.75	0.49	0.77
Precision	80	0.66	0.90	0.45	0.76	0.49	0.77
Precision	100	0.68	0.91	0.43	0.75	0.50	0.77
Precision	120	0.68	0.90	0.45	0.76	0.51	0.77
Precision	140	0.68	0.91	0.46	0.77	0.52	0.79
Precision	160	0.69	0.91	0.48	0.78	0.53	0.79
Recall	20	0.31	0.50	0.39	0.78	0.45	0.78
Recall	40	0.37	0.60	0.38	0.77	0.48	0.81
Recall	60	0.43	0.66	0.42	0.79	0.49	0.82
Recall	80	0.46	0.71	0.44	0.79	0.49	0.81
Recall	100	0.48	0.71	0.42	0.79	0.49	0.81
Recall	120	0.49	0.73	0.44	0.80	0.50	0.82
Recall	140	0.50	0.74	0.44	0.79	0.50	0.81
Recall	160	0.51	0.74	0.46	0.81	0.51	0.81
